# Cervical fracture dislocation without neurological abnormality: Rare case reports

**DOI:** 10.1016/j.ijscr.2024.109814

**Published:** 2024-05-29

**Authors:** Aliefio Japamadisaw, Aries Rakhmat Hidayat

**Affiliations:** Department of Orthopedic and Traumatology, Faculty of Medicine, Universitas Airlangga, Dr. Soetomo General Academic Hospital, Surabaya, Indonesia

**Keywords:** Cervical spinal cord injury, Cervical dislocation, Neurological deficit

## Abstract

**Introduction and importance:**

Traumatic lower cervical dislocation with spinal cord injury (SCI) can cause long-lasting dysfunction in many organ systems resulting in significant financial burden and functional disability. The patient may come with complete or incomplete neurological deficit. However, there is also possibility of no neurological deficit.

**Case presentation:**

This case reports presented two cases of a 68-year-old man and a 54-year-old man that came to the emergency department after a traffic accident and fell from a height. Surprisingly there was no neurological deficit found on both patients. The patient underwent emergency open reduction and posterior stabilization. Several months later, the neurological function was still excellent, and the pain was absent.

**Clinical discussion:**

Traumatic cervical dislocation without neurological deficit is rare. Enlargement of the spinal canal is significant when the vertebral body and the shattered posterior arch separate, which may play a protective role on the spinal cord. The neurological deficit did not happen in the first case due to a widening spinal canal. Still, in the second case, the patient's neurological condition remained excellent despite no disruption on the posterior arch after cervical dislocation.

**Conclusion:**

Neurological deficit may not occur in the cervical dislocation with disruption of the posterior arch due to the widening of the spinal canal. This injury should be treated properly to prevent other morbidities and even mortality. The posterior technique for stabilization gives various benefits, such as the safety and familiarity of the procedure and the high success rate.

## Highlights

• Cervical dislocation without neurological deficit
• Posterior stabilization in cervical dislocation
• Posterior arch fracture protects spinal cord from mechanical compression.

## 1 Introduction and importance

Traumatic cervical spine fractures account for one-third of all spine injuries. More than 50 % of these fractures occurred between C5-C7 [1]. This condition happened because the lower cervical is the transitional area to the thorax which is more rigid than the cervical vertebrae. Lower cervical fracture-dislocation also commonly causes spinal cord compression, resulting in significant financial burden and functional disability [2]. Traumatic spinal cord injury (SCI) is feared for its complications, as it can cause long-lasting dysfunction in many organ systems, creating an unstable breathing and hemodynamic condition, leading to a permanent change of function, which in the end causes an increasing number of morbidity and mortality to the patient. Regarding the increasing risk of unwanted problems caused by its complication, managing this kind of injury has to be done swiftly and accurately to prevent morbidity or mortality of the patient [3].

Traumatic spondylolisthesis of the cervical is less commonly occurring in trauma patients. The patient may come with the clinical features of complete or incomplete neurological deficit, and there is also a possibility of no neurological deficit. However, there is a high incidence of neurological deficit consequences after cervical trauma with 417 patients (64.9 %) during ten years of study in China [4]. Some reasons for the intact neurological examination are canal widening due to bi-pedicular or bi-laminar fractures or a combination. Spinal cord encroachment may be prevented by canal widening. This type of injury works as decompression to prevent nerve deficit due to spinal cord compression [5]. In this case series, we discuss the rare finding of traumatic cervical spine dislocation with and without posterior arch fracture in the absence of neurological deficit, which was successfully treated.

## 2 Case presentation

### 2.1 Case 1

A 68-years old man came to the emergency department due to a traffic accident 3 h before admission and he complained sharp pain on his neck around the posterior of C7. The Visual Analog Scale (VAS) evaluation from the patient was 7–8, indicating severe pain. The neurological examination of motor function, sensory function, and reflexes of this patient on the upper and lower extremity was normal. Pre-operative clinical evaluation by Neck Disability Index (NDI) according to the patient was 47 (complete disability).

**Fig. 1:**
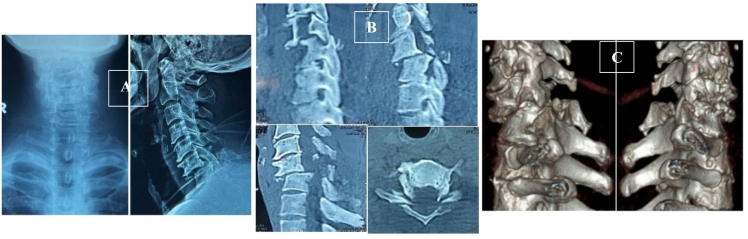
Pre-operative Cervical **(A)** plain x-ray and CT-Scan of **(B)** sagittal view, axial view, and **(C)** 3D view showed compression fracture on C7 and bilateral facet dislocation on vertebrae C6-C7 associated with bi-laminar fracture of C6.

Plain X-ray and CT-Scan showed compression fracture of C7 and bilateral facet dislocation on C6-C7 with 5.05 mm displacement associated with bi-laminar fracture of C6 (Fig. 1).

**Fig. 2:**
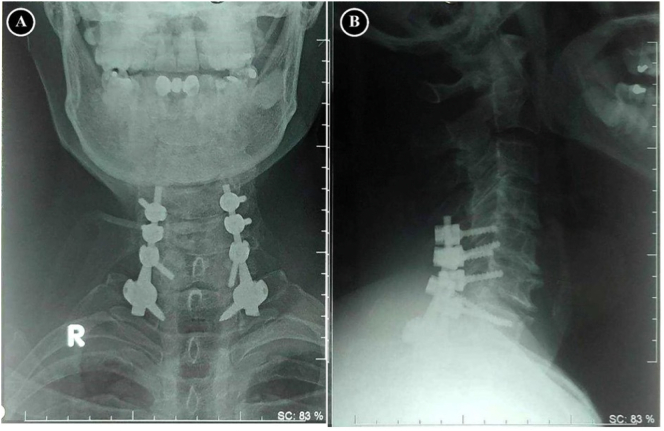
Postoperative radiography showed the realignment of the cervical spine in **(A)** Anterior View and **(B)** Lateral View, which stabilized using two lateral mass screws (C5-C6) and pedicle screws (C7-T1).

The emergency open reduction and posterior stabilization was performed (Fig. 2). Surgical treatment outcomes were evaluated at first month and sixth month after surgery. The VAS score at the first month was 1–2 and the sixth month was 0. NDI score improvement at the first month is 14 (mild disability) and 5 (mild disability) at the sixth month.

### 2.2 Case 2

**Fig. 3:**
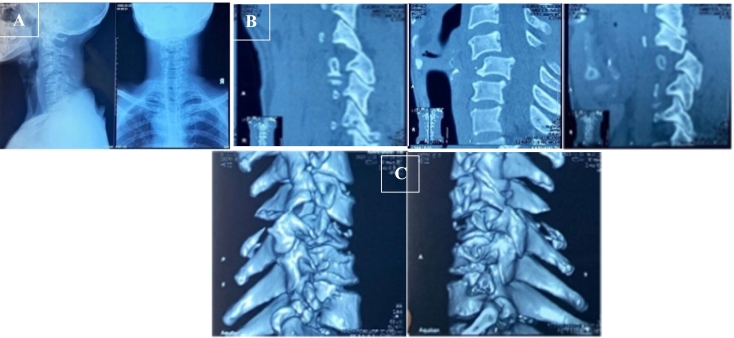
Pre-operative **(A)** Cervical Xray and CT-Scan of **(B)** Sagittal view and **(C)** 3D view showed bilateral facet dislocation on Vertebrae C4-C5, and there was no posterior arch fracture.

A 54-years old man came to the emergency department after falling from 2 m high. After the incident, he complained sharp pain on his neck. The VAS evaluation from the patient was 8–9. The NDI score on the pre-operative clinical evaluation was 45 (complete disability). Plain X-ray and CT-Scan found that there was a 35 % displacement of cervical vertebra C4-C5 about 6.1 mm (Fig. 3). We found also a bilateral facet dislocation in the cervical vertebrae C4-C5 without posterior arch fracture.

**Fig. 4:**
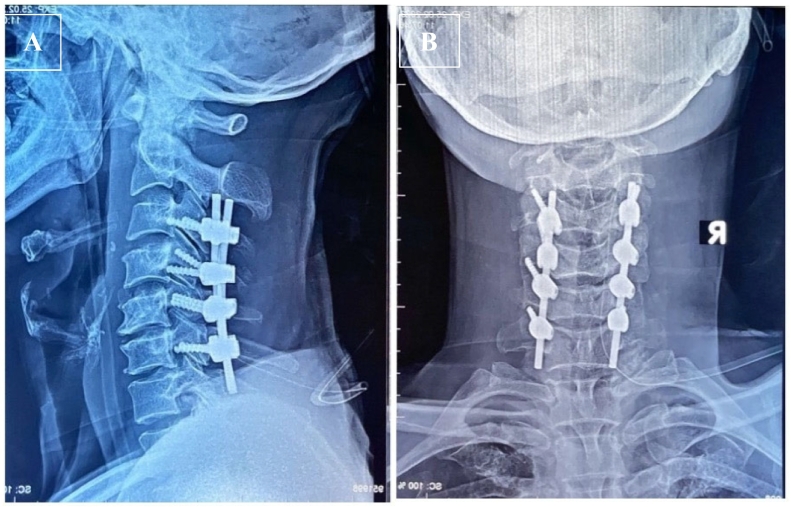
Postoperative radiography showed the realignment of the cervical spine in **(A)** Lateral View and **(B)** in Anterior View, which stabilized the cervical vertebrae from C3 until C6 using lateral mass.

The emergency open reduction and posterior stabilization was performed (Fig. 4). Clinical evaluation was done using VAS and NDI score by comparing the preoperative and postoperative condition. The VAS score at the first month was 1–2 and the sixth month was 0. NDI score improvement at the first month was 10 (mild disability) and 2 (no disability) at the sixth month.

## 3 Clinical discussion

Bilateral facet joint dislocation of the cervical spine is a severe injury that causes neurological deficit in 90 % of cases. Lower cervical fracture-dislocation causes spinal cord compression, significant financial burden, and functional disability. However, traumatic cervical spondylolisthesis without a neurological deficit is rare. In 1951, Perlman and Hawes were the first to report a case of cervical spondylolisthesis without the neurological deficit, and only a few patients with similar conditions since then. Cervical dislocation with posterior arch fracture causes canal enlargement, which may play a protective role on the spinal cord [[5], [6], [7]]. Previous study of the cervical fracture dislocation were describing their similar cases (Table 1). In our study, the first case was cervical dislocation with bilaminar fracture that respond to canal enlargement and preserve neurological function. On the other hand, the second case was cervical dislocation C4-C5 without separation of the posterior arch accompanied by 6.1 mm displacement with intact neurological function. While Ulbrich et al. study in Switzerland estimated that the difference between spinal canal and spinal cord diameter normally was 4.9 mm in sagittal view [8]. This may need further investigation and study about the cervical spinal actual normal dimensions in Asia. MRI is needed to evaluate the substantive condition of the spinal cord in our case.

**Table 1:** Cervical fracture dislocation data cases without neurological defect.

References	Age/sex	Fracture	Level	Timing of surgery	Surgery	Outcome
Ido et al., 2002 [7]	56/M	MPF + LF	C6-C7	10 days	Ant	Good
Acikbas and Gurkanlar, 2010 [9]	42/M	LMF + LF	C7-T1	5 days	Ant/post	Good
Ramieri et al., 2014 [5]	55/M	BPF	C6-C7	9 days	Post/Ant	Good
	38/M	BPF	C7-T1	7 days	Ant	Good
Our cases	68/M	BLF	C6-C7	36 h	Post	Good
	54/M	–	C4-C5	18 h	Post	Good

The anterior approach has been increasingly popular contradictory to the posterior approach. Proponents of the anterior approach argued that spinal cord compressions caused cervical dislocation were due to herniated disc or bone fragments which was from the anterior side. It allows surgeons to decompress the spinal canal by removing the bone fragments or herniated disc directly [9]. These injuries, which mainly affect the posterior bony and ligamentous structures, were most frequently stabilized using a posterior approach with rod and screw segmental fixation, in accordance with the fundamental principles of fracture management. The posterior technique for fixation has several advantages, including a high success rate for arthrodesis and the method's comfort and safety. In our cases, we performed posterior surgical approach in 18 h and 36 h respectively. However other studies showed no difference outcome in timing of the surgery neither its was performed early nor delayed [5,7,10].

Intraoperative Neurophysiological Monitoring (IONM) has been used to prevent or reduce substantial postoperative deficits by minimizing neurological injury during surgery and identifying crucial neural structures in the operative region. These modalities are including electromyography, motor evoked potentials (MEPs) and somatosensory evoked potentials (SSEPs) [11]. In our cases, the operative management were performed without IONM. However, some study showed the advantages of IONM to detect spinal cord injury during surgery [12]. The American Academy of Neurology (AAN) and the American Clinical Neurophysiology Society (ACNS) both advocated for the establishment of IONM using SSEPs and Tc-MEP as a reliable method of identifying an elevated risk of unfavorable outcomes, such as paraparesis, paraplegia, and quadriplegia, in spinal surgery [11]. However, in some studies showed that IONM signals are not reliable due to its false positive and negative rates are high [13,14]. This case series has been reported in accordance with the Surgical Case Report (SCARE) 2023 Criteria [15].

## 4 Conclusion

A neurological deficit may not occur in the cervical dislocation with disruption of the posterior arch due to the widening of the spinal canal. Still, in our case, the patient's neurological condition remains excellent even though there is no disruption on the posterior arch after cervical dislocation. This injury should be treated properly to prevent other morbidities and even mortality. The posterior technique for stabilization gives various benefits, such as the safety and familiarity of the procedure and the high success rate.

## Informed consent

Appropriate consent was obtained from all individual participants included in the study.

## Ethical approval

Regarding to the observational study of outcome in our case series, the ethical approval was waived by our institution. Moreover, due to multi-centre authors, our hospital academic institution could not provide the ethical clearance. However, the Author Form copies of informed consent are available for review by the Editor-in-Chief of this journal on request.

## Funding

N/A.

## Author contribution

Aliefio Japamadisaw involved in conceptualization, case presentation, data collection, main guidance for write up, and editing the manuscript.

Aries Rakhmat Hidayat involed in performing surgical technique, data collection, conceptualization, reviewing, and gave final approval for the manuscript.

## Guarantor

Aries Rakhmat Hidayat.

## Research registration number

N/A.

## Conflict of interest statement

The authors have no conflicts of interest to disclose.
